# The role of hydrogen sulphide signalling in macrophage activation

**DOI:** 10.1111/imm.13253

**Published:** 2020-10-06

**Authors:** Fei Sun, Jia‐Hui Luo, Tian‐Tian Yue, Fa‐Xi Wang, Chun‐Liang Yang, Shu Zhang, Xin‐Qiang Wang, Cong‐Yi Wang

**Affiliations:** ^1^ The Center for Biomedical Research Tongji Hospital, Tongji Medical College, Huazhong University of Sciences and Technology Wuhan China; ^2^ Institute of Organ Transplantation Tongji Hospital, Tongji Medical College, Huazhong University of Sciences and Technology Wuhan China; ^3^ Key Laboratory of Pulmonary Diseases of Health Ministry Tongji Hospital, Tongji Medical College, Huazhong University of Sciences and Technology Wuhan China; ^4^ Department of Respiratory and Critical Care Medicine Tongji Hospital, Tongji Medical College, Huazhong University of Sciences and Technology Wuhan China

**Keywords:** epigenetics, H_2_S, macrophage function, redox regulation, S‐sulphydration

## Abstract

Hydrogen sulphide (H_2_S) is the latest identified small gaseous mediator enabled by its lipophilic nature to freely permeate the biological membranes. Initially, H_2_S was recognized by its roles in neuronal activity and vascular relaxation, which makes it an important molecule involved in paracrine signalling pathways. Recently, the immune regulatory function of gasotransmitters, H_2_S in particular, is increasingly being appreciated. Endogenous H_2_S level has been linked to macrophage activation, polarization and inflammasome formation. Mechanistically, H_2_S‐induced protein S‐sulphydration suppresses several inflammatory pathways including NF‐κB and JNK signalling. Moreover, H_2_S serves as a potent cellular redox regulator to modulate epigenetic alterations and to promote mitochondrial biogenesis in macrophages. Here in this review, we intend to summarize the recent advancements of H_2_S studies in macrophages, and to discuss with focus on the therapeutic potential of H_2_S donors by targeting macrophages. The feasibility of H_2_S signalling component as a macrophage biomarker under disease conditions would be also discussed.

## Graphical abstract

Cellular H2S content is linked to macrophage functional status. H2S signalling regulates activation of macrophage. Targeting on H2S signalling modules represents a promising approach in treating macrophage‐related disorders.

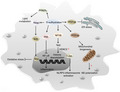

**Abbreviations**
3‐MST: 3‐mercaptopyruvate sulphur transferase
ATM: adipose tissue macrophage
CBS: cystathionine β‐synthase
CSE: cystathionine γ‐lyase
H_2_S: hydrogen sulphide
Hcy: homocysteine
oxLDL: oxidized low‐density lipoprotein

## Introduction

Gasotransmitters are a group of ubiquitous small gaseous signalling molecules, which mainly consist of nitric oxide (NO), carbon monoxide (CO) and hydrogen sulphide (H_2_S).^1^ Their lipophilic nature allows them to freely permeate through the biological membranes and to play an essential role in the regulation of cellular processes.^1^, ^2^ Indeed, dysregulation of gasotransmitter system is associated with numerous diseases ranging from neurological disorders to musculoskeletal abnormalities.^3^, ^4^, ^5^ Recently, encouraging results have further indicated a regulatory role for gasotransmitters in immune cells.^2^ In particular, macrophage, as the patrolling sentinel in the immune system, is extensively regulated by these gaseous mediators.^6^

Upon activation, the classically activated (M1) macrophages upregulate the expression of inducible nitric oxide synthase (iNOS), and catalyse the transformation of L‐arginine to NO. Elevated NO along with the production of reactive nitrogen species is indispensable for the optimal antimicrobial activity and the secretion of inflammatory cytokines such as IL‐6, TNF‐α and interferons.^7^, ^8^ On the other hand, the alternatively activated (M2) macrophages highly express the hallmark enzyme Arginase1 (Arg1), which outcompetes the activity of iNOS on l‐arginine availability and reduces the NO production.^9^ Therefore, the fluctuation of NO metabolism serves as a key molecular switch for control of macrophage function to dynamically regulate the initiation or resolution of an inflammatory response. In contrast to NO, CO, a haem metabolism product produced by the haem oxygenase 1‐3 (HO 1‐3), attenuates macrophage activation, and therefore, HO‐1 overexpression in myeloid lineages favours M2 programme in macrophages and implies better outcome in liver transplant patients.^10^ Consistently, HO‐1 deficiency leads to increased M1 macrophages along with enhanced inflammatory infiltration following ischaemia–reperfusion injury.^10^ Similarly, CO suppresses lipopolysaccharide (LPS)‐induced macrophage activation and induces the secretion of IL‐10, which involves its effect on the activation of mitogen‐activated protein kinase kinase 3 (MKK3).^11^

H_2_S, the latest identified gasotransmitter, was first recognized as a smelly and environmental toxic gas.^12^ Past two decades of studies revealed that H_2_S can be generated endogenously and work as an autocrine signalling molecule.^3^, ^13^, ^14^ In mammals, three enzymes including cystathionine γ‐lyase (CSE), cystathionine β‐synthase (CBS) and the 3‐mercaptopyruvate sulphur transferase (3‐MST) are responsible for H_2_S generation.^15^, ^16^ Specifically, CSE and CBS catalyse de‐sulphydration of cysteine to generate H_2_S, while MST induces H_2_S production by regulating the enzymatic activity of cysteine aminotransferase.^17^, ^18^ The essential role of H_2_S signalling in T‐cell biology has been well addressed, in which ablation of CBS and CSE leads to impaired T‐cell activation and proliferation.^19^ Mice deficient in *CBS* also manifest reduced regulatory T cells along with massive inflammatory infiltration, which could be reversed by H_2_S donor supplementation.^20^

Interestingly, unlike its effect on T cells, in macrophages, H_2_S signalling is clearly anti‐inflammatory in a variety of interesting ways. It seems that H_2_S actively impact macrophage on its activation, polarization and inflammasome formation through distinct mechanistic pathways. Particularly, macrophages likely also set the threshold for the activation of H_2_S signalling under various stimuli. Herein, we aim to summarize the regulatory mechanisms underlying H_2_S signalling and discuss with focus for the impact of H_2_S signalling on the regulation of macrophage functionality. We also discuss the potential that the cellular H_2_S content and the key H_2_S metabolic enzymes serve as ideal biomarkers to indicate distinct macrophage activation status.

## The regulatory mechanisms underlying H_2_S signalling

**Figure 1:**
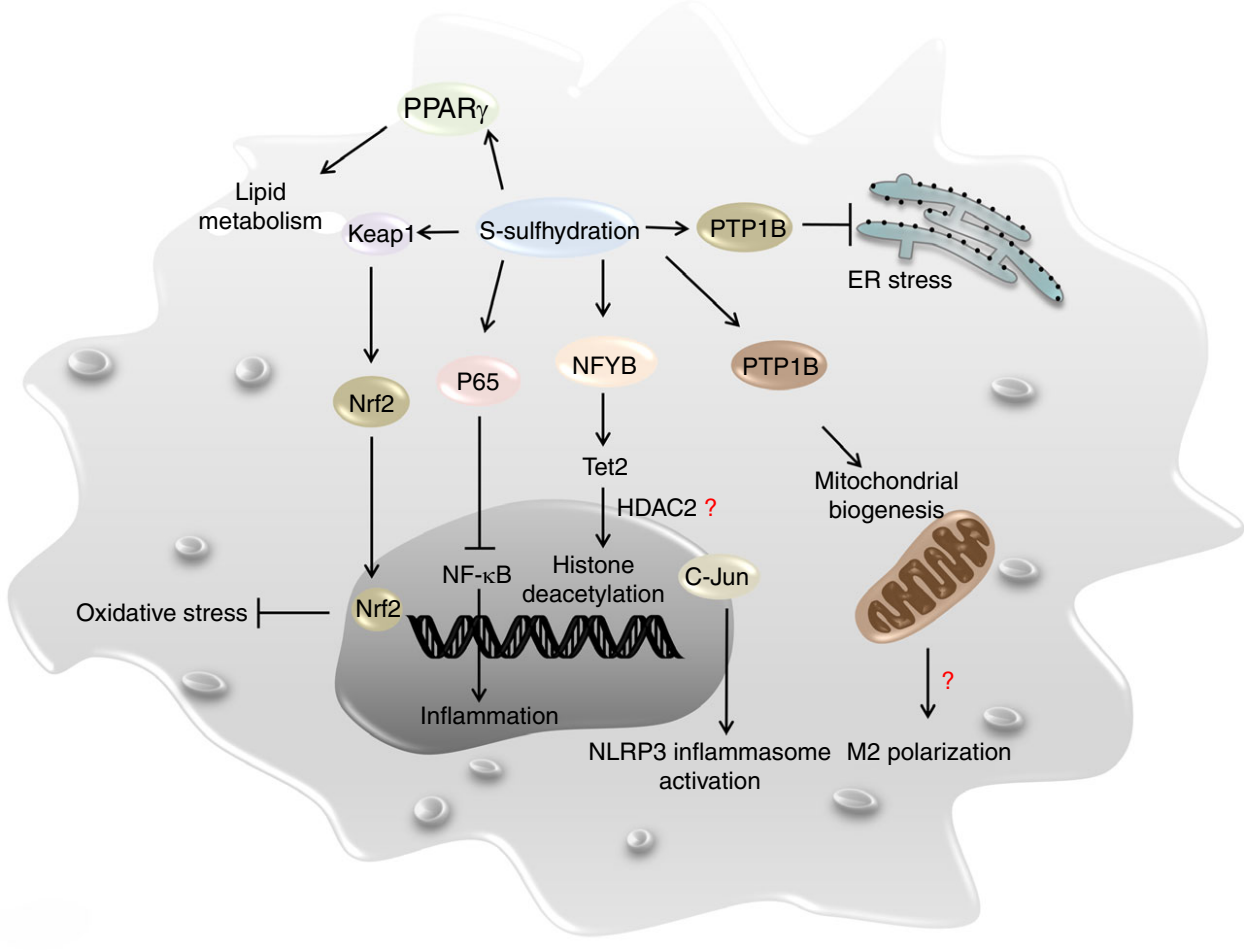
Potential regulatory mechanisms underlying H_2_S signalling. The mechanisms underlying H_2_S signalling in the regulation of macrophage function presumably involve direct mediation of protein S‐sulphydration, cellular redox homeostasis and epigenetic chromatin remodelling.

H_2_S signalling plays a critical regulatory role in diverse immune responses, which involves H_2_S‐induced protein S‐sulphydration, cellular redox homeostasis and epigenetic chromatin remodelling (Fig. 1). In this section, we briefly summarize the above regulatory mechanisms underlying H_2_S signalling.

### Protein S‐sulphydration

H_2_S‐induced protein S‐sulphydration is a novel post‐translational modification occurring on specific cysteine (Cys) residues of target proteins, by which it regulates the biological activity of targeted proteins. It is noteworthy that S‐sulphydration of key enzymes, receptors and transcriptional factors contributes a major part to H_2_S signalling and its regulatory function. Kir6.1, a subunit of ATP‐sensitive potassium channels (K_ATP_), is S‐sulphydrated at Cys43, which promotes K_ATP_ channel activity and improves vasodilation.^21^ Other ion channels such as voltage‐activated calcium channels, and transient receptor potential channel proteins TRPV6 and TRPV4, were also suggested to be S‐sulphydrated, thereby regulating calcium flux.^22^, ^23^ Together, these events perfectly explain the effect of endogenous H_2_S and exogenous H_2_S donors on vascular relaxation.

Metabolic reprogramming and stress responses including oxidative stress and endoplasmic reticulum (ER) stress are critical regulators in immune cells and their fate decision. Other than the well‐known role in cardiovascular system, H_2_S‐mediated protein S‐sulphydration also engages in the metabolic processes and cellular stress responses. S‐sulphydration of peroxisome proliferator‐activated receptor‐γ (PPARγ) at Cys139 enhances its DNA binding activity and the subsequent expression of adipogenic genes, thus increasing glucose uptake and lipid metabolism.^24^ Additionally, H_2_S promotes the activities of PPARγ coactivator‐related protein (PPRC), alpha subunit of ATP synthase (ATP5A1) and interferon regulatory factor 1 via S‐sulphydration, by which it stimulates mitochondrial biogenesis and protects against mitochondrial dysfunction.^25^, ^26^, ^27^ P66Shc is an upstream activator of mitochondrial redox signalling, and studies suggested that H_2_S protects neuronal cells against stress‐induced senescence by inducing its S‐sulphydration at Cys59 residue.^28^ H_2_S also induces Keap1 S‐sulphydration (Cys151, Cys226 and Cys613) to promote the dissociation of Keap1‐Nrf2 complex, thereby releasing Nrf2 to transcribe the expression of antioxidant genes ^29^, ^30^. Similarly, PTP‐1B is a protein tyrosine phosphatase related to the deactivation of protein kinase RNA‐like ER kinase (PERK), while H_2_S mediates PTP‐1B S‐sulphydration at Cys215 to inhibit its enzymatic activity, thereby activating PERK pathway to alleviate ER stress.^31^

It is worthy of note that some immune regulatory molecules are the direct targets for H_2_S‐induced S‐sulphydration. For example, S‐sulphydration of nuclear transcription factor Y subunit beta (NFYB) at Cys105 increases the transcription of the ten‐eleven translocation (Tet) genes.^32^ Tet1 and Tet2 in turn bind to the regulatory regions within the *Foxp3* gene to maintain the hypomethylation status of its promoter and the conserved non‐coding sequence 2 (CNS2) region, thereby ensuring Foxp3 expression and the stability of Treg cell lineage.^20^ Similarly, S‐sulphydration of the free thiol group Cys38 in p65 inhibits NF‐κB activity in macrophages.^33^ Moreover, S‐sulphydration of c‐Jun at Cys269 attenuates hydrogen peroxide (H_2_O_2_)‐induced NLRP3 inflammasome activation and reduces IL‐1β production in macrophages.^34^

### Cellular redox homeostasis

Theoretically, most H_2_S can dissolve in surface water and dissociate into HS^−^ under normal circumstances (37°, pH = 7.4),^35^ and HS^−^ in turn could serve as a powerful one‐electron chemical reductant to scavenge ROS. In reality, however, the physiological concentration of H_2_S is at the sub‐micromolar level,^36^ which is too low for H_2_S to act as a direct antioxidant. Nonetheless, low concentration of endogenous H_2_S can exert potent antioxidant effects in alternative manners. Specifically, other than the aforementioned Keap1 S‐sulphydration‐mediated pathway, hypoxia‐inducible factor 1α (HIF‐1α) also serves as another important molecule downstream of H_2_S signalling.^37^ Studies in THP‐1 cells, a human macrophage cell line, revealed that H_2_S induces HIF‐1a nuclear translocation to enhance the expression of glucose transporter GLUT1 along with the abrogation of its pro‐inflammatory effect.^37^ Consistently, it was also found that H_2_S could activate the antioxidant Nrf2/HO‐1 pathway by stimulating the p38 mitogen‐activated protein kinase (MAPK) activity.^37^ Therefore, H_2_S has been found to attenuate LPS‐induced acute lung injury by reducing oxidative and nitrative species,^38^ and H_2_S administration improves glutathione (GSH) level along with alleviated lipid peroxidation and allergic lung inflammation.^39^ Collectively, as a negative regulator in cellular redox homeostasis, H_2_S exhibits anti‐inflammatory potency amid stress‐related inflammatory disorders.

### Epigenetic chromatin remodelling

Another critical mechanism underlying H_2_S signalling is that H_2_S also manifests a remarkable capacity to regulate epigenetic chromatin remodelling. Apart from the above‐introduced NFYB‐Tet pathway, which mediates DNA demethylation of the *Foxp3* regulatory regions in Treg cells, H_2_S exhibits high potency to remodel chromatin structure through regulation of histone modifications in macrophages.

The Jumonji domain‐containing protein 3 (JMJD3) is a histone 3 Lys27 (H3K27) demethylase and plays a critical role in chromatin remodelling.^40^ There is evidence that LPS upregulates CSE expression in macrophages in a mouse model with septic shock, and enhanced CSE in turn inhibits JMJD3 expression to increase H3K27me3 levels, thereby attenuating LPS‐mediated inflammatory response.^41^ Studies in macrophages further noted that H_2_S is capable of suppressing histone acetylation at the IL‐6 and TNF‐α promoter, by which it inhibits chromatin openness to repress the transcription of inflammatory cytokines following LPS stimulation.^42^ Although no direct evidence shows the existence of H_2_S‐NFYB‐Tet pathway in macrophage, Tet2 resolves macrophage inflammatory response by recruiting HDAC2 and deacetylating permissive histone markers in the IL‐6 promoter, the mechanism of which is DNA methylation‐independent and quite different from what happens in Treg cells.^43^ These results suggest that the CSE/H_2_S signalling could be vital to prevent uncontrolled macrophage inflammatory responses via epigenetic machineries.

Heretofore, the major mechanism underlying H_2_S signalling is likely attributed to the S‐sulphydration of substrate proteins (Table 1). Moreover, the impact of H_2_S signalling on the regulation of redox homeostasis and chromatin remodelling seems independent of S‐sulphydration, but additional studies would be necessary to fully address this issue. It should be also important to keep in mind that characterization of additional unidentified S‐sulphydration proteins would help to completely clarify the regulatory mechanisms.

**Table 1:** Potential S‐sulphydration targets relevant to macrophage regulation

Potential target	Modification site	Major cell types	Biological consequence	Reference
P65	Cys38	Macrophage	Inhibiting NF‐κB activity	^33^
c‐Jun	Cys269	Macrophage	Attenuating inflammasome activation and IL‐1β production	^34^
Keap1	Cys151, Cys226, Cys613	Fibroblast	Dissociation of Keap1‐Nrf2 complex; antioxidative response	29, 30
PTP1B	Cys215	293T cell	Alleviating ER stress	^31^
PPARγ	Cys139	Adipocyte	Enhancing DNA binding activity of PPARγ, increasing lipid metabolism	^24^
NFYB	Cys105	Regulatory T cell	Promoting the transcription of Tet1/2	20, 32

Keap1, Kelch‐like ECH‐associated protein 1; NFYB, nuclear transcription factor Y subunit beta; PTP1B, protein tyrosine phosphatase 1B; Tet, tet methylcytosine dioxygenase 2.

## H_2_S signalling in maintaining the M1/M2 homeostasis in macrophages

As described earlier, macrophages display different functional phenotypes depending on their residing environmental milieu. For simplicity, they are classified into two distinct subtypes: one is classically activated (M1) macrophages, and the other is alternatively activated (M2) macrophages. LPS and IFN‐γ induce the generation of M1 macrophages, which then augment the production of pro‐inflammatory cytokines. In contrast, M2 macrophages are elicited by glucocorticoids or type II cytokines such as IL‐4, IL‐13 and IL‐10. M2 macrophages are responsible for wound healing, tissue repair and the resolution of inflammation, thus generally regarded as an anti‐inflammatory cell type.

Recent studies provided compelling evidence that H_2_S signalling is implicated in dictating macrophage polarizations. Initially, the endogenous H_2_S was found to attenuate LPS‐induced oxidative stress and inflammatory damage by inhibiting NOX4‐ROS signalling pathway in macrophages.^44^ GYY4137, a novel H_2_S‐releasing molecule, was confirmed to inhibit rat endotoxic shock and mucosal wound through abrogating M1 programme in macrophages.^45^, ^46^ Similarly, FW1256, another slow‐releasing H_2_S donor, was further noted to exhibit anti‐inflammatory properties by reducing the production of inflammatory mediators such as TNF‐α, IL‐6, PGE2, IL‐1β, COX‐2 and NO in macrophages.^47^ Subsequent mechanistic studies demonstrated that NaHS promotes macrophage M2 polarization by enhancing mitochondrial biogenesis and fatty acid oxidation (FAO).^48^ Similar results were also observed in the central nervous system, in which H_2_S exerts neuroprotection against hypoxia‐induced neurotoxicity through induction of M2 programme in microglia cells by inhibiting iNOS, NF‐κB, ERK and p38 MAPK signalling pathways.^49^ Therefore, H_2_S signalling serves as a critical regulatory mechanism to maintain the homeostatic M1/M2 balance in the setting of inflammatory resolution.

## H_2_S signalling in macrophage activation and inflammasome formation

**Figure 2:**
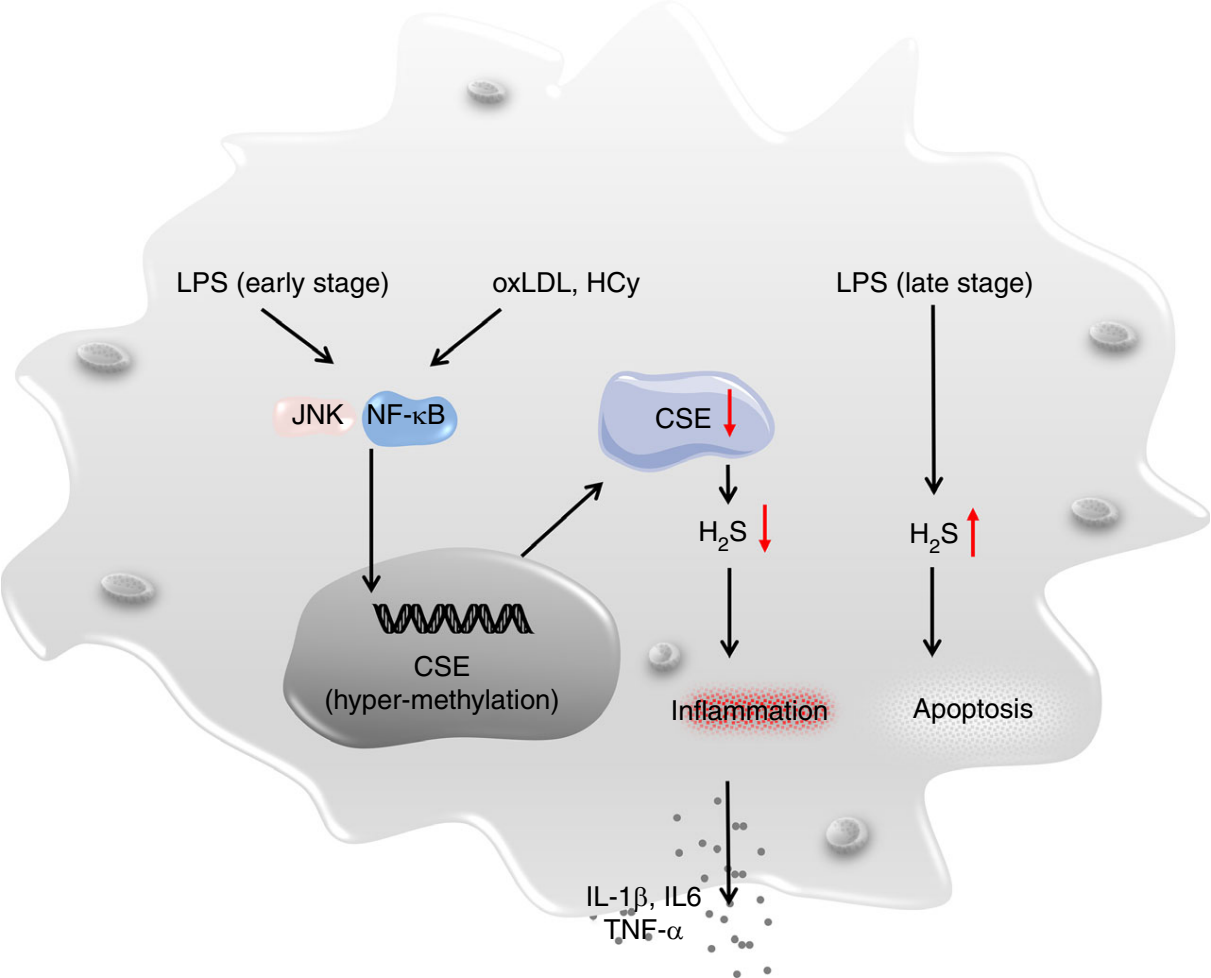
H_2_S signalling regulates macrophage functionality for the initiation and resolution of an inflammatory response. Upon stimulation (e.g. LPS and oxLDL), H_2_S production is shut down at the early stage to facilitate pro‐inflammatory cytokine secretion, while at the late stage, the H_2_S content becomes increased for induction of those mission‐completed macrophages to undergo apoptosis. Alerted H_2_S signalling would lead to the development of immune or metabolic disorders. LPS, lipopolysaccharide; oxLDL, oxidized low‐density lipoprotein.

It was noted that LPS‐stimulated macrophages and adipose tissue macrophages (ATMs) derived from diet‐induced obese mice manifest lower intracellular concentration of H_2_S,^50^ suggesting that depletion of macrophage H_2_S content occurs during both acute (LPS‐induced) and chronic (obesity) inflammatory conditions. Indeed, oxidized low‐density lipoprotein (oxLDL) induces the CSE promoter to undergo DNA hypermethylation in macrophages, leading to attenuated CSE transcription and H_2_S production in favour of inflammatory responses,^51^ which involves the activation of JNK/NF‐κB signalling.^52^ Similarly, homocysteine (HCy) induces DNA hypermethylation in the CSE promoter in macrophages, through which it exaggerates inflammation by inhibiting CSE‐H_2_S signalling^53^ (Fig. 2).

In line with above observations, a time‐dependent change of H_2_S content in macrophages was found following activation. A decrease of H_2_S level in murine macrophages following 24 hr of LPS or IFN‐γ stimulation was observed (early phase), but the H_2_S content was restored to normal level after 48 hr of stimulation (late phase), which was associated with the feedback regulation between CBS and CSE.^54^ It is worthy of note that H_2_S production was correlated with LPS‐induced macrophage late‐stage apoptosis, which could be blocked by the addition of H_2_S inhibitor.^55^ Therefore, it is possible that sustained LPS stimulation renders macrophages that undergo apoptosis through the production of H_2_S (Fig. 2).

Macrophages not only sense exogenous pathogen‐associated molecular patterns (e.g. LPS) derived from micro‐organisms, but also respond to endogenous stimuli. The most commonly seen endogenous insults originate from harmful metabolites, such as excessive free fatty acids (FFAs) and oxLDL. Interestingly, these metabolites alone could lead to abnormal macrophage activation, while they could also serve as the second signals essential for inflammasome formation. Inflammasome is a complex of proteins found in macrophages that regulates the activation of caspase enzymes and induces the secretion of pro‐inflammatory cytokines (e.g. IL‐1β and IL‐18). Importantly, recent studies demonstrated that both exogenous and endogenous H_2_S inhibit NLRP3 inflammasome activation and reduce inflammatory cytokine production in macrophages.^56^ In particular, upregulation of H_2_S content by treating the cells with NaHS reduces the expression level of inflammasome‐associated proteins such as TXNIP, NLRP3, ASC and caspase‐1 by inhibiting thioredoxin‐interacting protein–NLRP3 (TXNIP‐NLRP3) signalling pathway.^57^ Taken together, H_2_S signalling not only directly represses macrophage activation, but also inhibits inflammasome formation, thereby attenuating inflammatory responses.

## The therapeutic potential for targeting H_2_S signalling in macrophages

Macrophages are critical participants in the immune system, which are involved in innate immunity and also help to recruit other immune cells for adaptive immune responses. Macrophages can be found essentially in all tissues, and their dysfunction is linked to a variety of diseases. Dysregulation of macrophages is related to various diseases ranging from infection to metabolic disorders, wherein H_2_S donors exhibit significant therapeutic potential.

It has been well recognized that enhanced H_2_S signalling in macrophages abrogates the progression of septic shock,^45^ a severe inflammatory disorder caused by bacterial infection and now faces up with limited therapeutics in clinic. Microglia, a specialized macrophage in the nervous system, is involved in the pathogenesis of Alzheimer’s and Parkinson’s disease. Given the role of H_2_S signalling in the resolution of neuronal inflammation,^58^, ^59^, ^60^ H_2_S donors are proven to be effective in numerous neuronal disorders.^49^, ^60^ Similarly, as H_2_S reduces FFAs and oxLDL‐induced metabolic stress and inflammasome formation, H_2_S donors could inhibit foam cell formation and attenuate the release of pro‐inflammatory cytokines, thus leading to the amelioration of arterial atherosclerosis and other inflammasome‐associated diseases such as DSS‐induced colitis.^57^, ^61^, ^62^ MicroRNA‐186 (miR‐186) plays an important role in atherosclerotic diseases. Mechanistic study revealed that miR‐186 directly binds to the 3’‐UTR of CSE and destabilizes the mRNA transcripts. As a result of decreased CSE‐H_2_S axis, the human macrophages take up more lipids and become pro‐inflammatory.^63^ Exogenous administration of H_2_S donor NaHS or GYY4137 decreases the inflammatory cytokine secretion, prohibits lipid accumulation in macrophages and down‐tunes the expression of chemokine receptors (CX3CR1 in particular), thus demonstrating the effectiveness in atherosclerosis treatment.^64^, ^65^ As aforementioned, intracellular concentration of H_2_S was lower in ATMs of obese mice, and not surprisingly, exogenous supplementation of H_2_S donors could curb the development of obesity and the subsequent metabolic syndromes.^50^

Alternatively activated M2 macrophages substantially participate in inflammation resolution and tissue repair. Given the role of H_2_S signalling played in M2 macrophages, it is not surprising that H_2_S would play a pivotal role in myocardial infarction (MI) and wound healing. Studies showed that H_2_S promotes macrophage migration towards the infracted area at the early stage, then induces M2 polarization by enhancing mitochondrial biogenesis and FAO, the two steps of which cooperatively accelerates the post‐MI recovery.^48^, ^66^ During the wound healing process, the local H_2_S content was found to significantly reduce amid injured tissue granulation. Replenishment of H_2_S inhibits macrophage activation and improves wound healing in both oral mucosal wound model and diabetic wound model.^46^, ^67^ In situ induction of M2 macrophages by employing the novel H_2_S‐releasing hydrogel greatly improves wound healing process, which displays a promising translational potential.^68^ Together, these results support that targeting H_2_S signalling in macrophages could be a viable approach to fight against immune and metabolic disorders in clinical settings and to restore tissue homeostasis upon trauma.

## Concluding remarks and perspectives

It would be important to note that the relationship between H_2_S signalling and macrophage functionality is reciprocal and dynamic. H_2_S actively modulates macrophage activation, polarization and inflammasome formation (Fig. 1), and macrophages in turn influence the intrinsic H_2_S synthetic machinery following external stimuli. Specifically, upon LPS stimulation, H_2_S production is shut down at the early stage to facilitate pro‐inflammatory cytokine secretion, while at the late stage, the H_2_S content becomes increased for induction of those mission‐completed macrophages to undergo apoptosis. This complex feedback loop underpins the multifaceted function of macrophages, which reflects a fine control of macrophage‐mediated immune response (Fig. 2).

Generally, H_2_S induces S‐sulphydration of key signalling molecules, such as p65 and c‐Jun, to impact on NF‐κB pathway and canonical NLRP3 inflammasome formation, while H_2_S also regulates cellular redox homeostasis and chromatin remodelling to affect macrophage function. However, we cannot exclude the possibility that additional unrecognized S‐sulphydrated proteins could be also engaged in H_2_S signalling. As for the regulation of cellular redox homeostasis, it is intriguing that H_2_S‐induced S‐sulphydration shares great similarity as GSH‐mediated S‐glutathionylation,^69^, ^70^ and both of which even possess the same substrate, PTP1B.^31^, ^71^ There is evidence that S‐glutathionylation regulates redox homeostasis,^72^ and a typical example is MKP1, which has been verified to be a substrate for S‐glutathionylation.^73^ It is therefore plausible that H_2_S could either directly mediates MKP1 S‐sulphydration to regulate macrophage redox homeostasis, or indirectly influences MKP1 S‐glutathionylation by elevating GSH levels, which could perfectly explain the inhibitory effect of H_2_S on MAPK signalling.

Macrophages demand distinct intracellular metabolic pathways depending on their functional state. The activation of M1 macrophages by LPS or IFN‐γ is associated with higher glycolysis along with attenuated tri‐carboxylic acid (TCA) cycle and mitochondrial oxidative phosphorylation (OXPHOS).^74^ In contrast, M2 macrophages require higher mitochondrial biogenesis, fatty acid uptake and FAO.^75^, ^76^ Collectively, those discoveries support that H_2_S‐mediated metabolic reprogramming finely controls the initiation and resolution of an inflammatory response.^48^ Therefore, a better understanding of the role for H_2_S signalling in macrophages would demonstrate great potential to develop therapies against either acute or chronic inflammatory responses in clinical settings of patients with immune or metabolic disorders. Indeed, some commonly prescribed drugs have already been indicated to affect endogenous H_2_S signalling pathway. For example, statins are able to modulate H_2_S metabolism^77^, ^78^ in the cardiovascular system, while the well‐known antidiabetic drug, metformin, could promote H_2_S production by elevating CSE.^79^ These discoveries prompt us to rescrutinize the ‘new function of old drugs’ while pursuing for novel H_2_S regulating compounds in the future investigations.

## Disclosures

The authors declare no conflict of interest.

## Data Availability

Data availability is not applicable.
